# More Exercise Is Linked to Less Loneliness in Adult Day Health Attendees

**DOI:** 10.1093/geroni/igaa057.1025

**Published:** 2020-12-16

**Authors:** Wingyun Mak

**Affiliations:** The New Jewish Home, New York, New York, United States

## Abstract

There is a growing evidence that more physical activity is linked to less loneliness (Cigna, 2018; Pels & Kleinert; 2016). It is less clear whether this relationship persists in older adults who are attending adult day health services and often have multiple comorbidities. In this study, we examined whether spending more time exercising would significantly predict whether adult day health attendees reported loneliness. We used UAS-NY data from a sample of 221 adult day health attendees from 2019 to early 2020 who scored five or greater on the Nursing Facility Level of Care Index, which is a score derived from assessments of cognition, communication and vision, mood and behavior, functional status, continence, and nutritional status. A logistic regression showed that after controlling for demographic variables, cognition, health, and quality of family relationships, participants were less likely to report loneliness if they had been attending adult day health services for at least six months or more (B=.86, p<.05), spent less time alone (B=-.45, p<.05), and exercised more (B=.47, p<.05; χ2(3)=15.6, p=.001). These results suggest that in addition to participating in adult day services and spending more time with others, exercising may have an impact on the experience of loneliness.

